# Impact of Intratracheal Administration of Polyethylene Glycol-Coated Silver Nanoparticles on the Heart of Normotensive and Hypertensive Mice

**DOI:** 10.3390/ijms24108890

**Published:** 2023-05-17

**Authors:** Abderrahim Nemmar, Suhail Al-Salam, Yaser E. Greish, Sumaya Beegam, Nur E. Zaaba, Badreldin H. Ali

**Affiliations:** 1 Department of Physiology, College of Medicine and Health Sciences, United Arab Emirates University, Al Ain P.O. Box 17666, United Arab Emirates; sumayab@uaeu.ac.ae (S.B.); elenazaaba@uaeu.ac.ae (N.E.Z.); 2 Zayed Center for Health Sciences, United Arab Emirates University, Al Ain P.O. Box 17666, United Arab Emirates; suhaila@uaeu.ac.ae; 3 Department of Pathology, College of Medicine and Health Sciences, United Arab Emirates University, Al Ain P.O Box 17666, United Arab Emirates; 4 Department of Chemistry, College of Science, United Arab Emirates University, Al Ain P.O. Box 17551, United Arab Emirates; y.afifi@uaeu.ac.ae; 5 Department of Pharmacology and Clinical Pharmacy, College of Medicine and Health Sciences, Sultan Qaboos University, Muscat 123, Oman; alibadreldin@hotmail.com

**Keywords:** silver nanoparticles, polyethylene glycol, coating, hypertension, cardiotoxicity

## Abstract

Silver nanoparticles are widely used in various industrial and biomedical applications; however, little is known about their potential cardiotoxicity after pulmonary exposure, particularly in hypertensive subjects. We assessed the cardiotoxicity of polyethylene glycol (PEG)-coated AgNPs in hypertensive (HT) mice. Saline (control) or PEG–AgNPs (0.5 mg/kg) were intratracheally (i.t.) instilled four times (on days 7, 14, 21, and 28 post-angiotensin II or vehicle [saline] infusion). On day 29, various cardiovascular parameters were evaluated. Systolic blood pressure and heart rate were higher in PEG–AgNPs-treated HT mice than in saline-treated HT or PEG–AgNPs-treated normotensive mice. The heart histology of PEG–AgNPs-treated HT mice had comparatively larger cardiomyocyte damage with fibrosis and inflammatory cells when compared with saline-treated HT mice. Similarly, the relative heart weight and the activities of lactate dehydrogenase and creatine kinase-MB and the concentration of brain natriuretic peptide concentration were significantly augmented in heart homogenates of HT mice treated with PEG–AgNPs compared with HT mice treated with saline or normotensive animals exposed to PEG–AgNPs. Similarly, the concentrations of endothelin-1, P-selectin, vascular cell adhesion molecule-1, and intercellular adhesion molecule-1 in heart homogenates were significantly higher than in the other two groups when HT mice were exposed to PEG–AgNPs. Markers of inflammation and oxidative and nitrosative stress were significantly elevated in heart homogenates of HT mice given PEG–AgNPs compared with HT mice treated with saline or normotensive animals exposed to PEG–AgNPs. The hearts of HT mice exposed to PEG–AgNPs had significantly increased DNA damage than those of HT mice treated with saline or normotensive mice treated with AgNPs. In conclusion, the cardiac injury caused by PEG–AgNPs was aggravated in hypertensive mice. The cardiotoxicity of PEG–AgNPs in HT mice highlights the importance of an in-depth assessment of their toxicity before using them in clinical settings, particularly in patients with pre-existing cardiovascular diseases.

## 1. Introduction

Silver nanoparticles (AgNPs, ≤100 nm in size) are one of the most commonly used nanomaterials in various applications, such as medical materials, antibiotics, and appliances due to their large surface area and proportion of surface atoms. The advantages of these nanoparticles have been widely publicized. However, their potential toxicity has received significantly less attention [1,2].

Human exposure to these nanoparticles can occur through skin penetration, ingestion, or inhalation during the manufacturing or use of AgNPs [2,3]. AgNPs have been reported to pass through the alveolar–capillary barrier, reach the blood, and distribute to numerous organs, including the heart, after being inhaled due to their small size [2].

Coating with different agents (e.g., polyvinylpyrrolidone [PVP] or citrate) is currently used to make potentially safe preparations of AgNPs to preserve biological activity, improve stability, and reduce toxicity [2,4]. These agents stabilize the nanoparticles and prevent oxidative dissolution [5]. Similarly, polyethylene glycol (PEG) coating is used to neutralize the charge on the nanoparticles’ surfaces and stabilize them via steric interference [4]. The latter coating on the nanoparticles has been reported to reduce their reactivity and improve the degree of penetration through the mucus layer, bolstering the case for its use in nanomedicine [6]. However, understanding the efficacy and safety of PEG–AgNPs on human health is still limited. An in vitro study has reported that AgNPs coated with PEG or citrate are cytotoxic to human keratinocytes HaCaT cells [7]. Conversely, another in vitro study has shown that PEG coating is able to shield nanoparticles from the immune system, reducing their toxic effects [6]. Moreover, a recent in vivo investigation has reported that thrombosis is aggravated in hypertensive mice exposed to PEG–AgNPs compared with normotensive mice [8]. However, as far as we are aware, no study has investigated the cardiotoxicity and underlying mechanisms of action of PEG–AgNPs after pulmonary exposure, particularly in hypertensive animal models.

There is a robust association between lung exposure to particulate matter and cardiovascular toxicity [3]. Particulate air pollution has been shown in epidemiological and clinical studies to have a greater impact on subjects with pre-existing cardiovascular diseases, such as hypertension [3]. Nonetheless, research on the effects of AgNPs on cardiac homeostasis in hypertensive animals is limited. A recent ex vivo study found that administering AgNPs as a bolus to isolated heart Langendorff preparation of spontaneously hypertensive rats induced vasoconstriction, increased cardiac contractility, and caused oxidative stress [9]. Nevertheless, in vivo investigations of the cardiotoxicity of pulmonary administration of PEG–AgNPs in experimental hypertension has not been previously reported.

The current study hypothesized that the cardiotoxicity of PEG–AgNPs would be aggravated in hypertensive mice compared with normotensive mice. Therefore, this study aims to evaluate the effect of pulmonary exposure to PEG–AgNPs on blood pressure, heart histology, and various markers of cell adhesion molecules, proinflammatory cytokines, oxidative and nitrosative stress, and DNA damage in a hypertensive mouse model.

## 2. Results

### 2.1. Characterization of PEG–AgNPs

**Figure 1:**
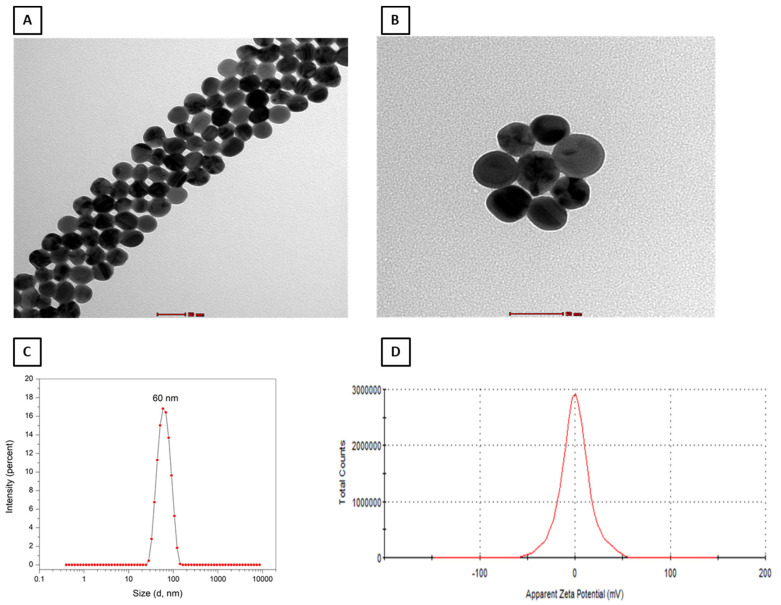
Transmission electron microscope analysis of polyethylene glycol silver nanoparticles (PEG–AgNPs) (**A**,**B**). Representative images showing size distribution (**C**) and zeta potential analysis (**D**) PEG–AgNPs.

As previously reported [8], TEM size analysis of AgNPs confirmed the presence of individual spherical nanoparticles of approximately 40 nm in size with a homogeneous size distribution of agglomerated nanoparticles in this study (Figure 1A,B). On the other hand, our zeta sizer measured an average size of 57.6 nm with a unimodal homogeneous size distribution (Figure 1C). Moreover, the zeta potential examination of these PEG–AgNPs revealed a nearly zero charge, indicating charge neutrality (Figure 1D).

### 2.2. Systolic Blood Pressure (SBP) and Heart Rate (HR)

**Figure 2:**
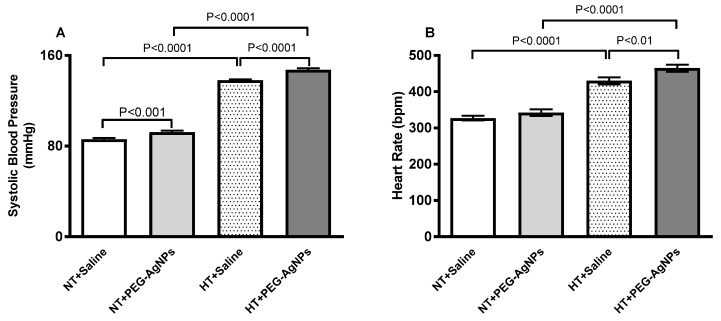
Systolic blood pressure (**A**) and heart rate (**B**) in normotensive (NT) and hypertensive (HT) mice after repeated intratracheal (i.t.) instillation of saline or polyethylene glycol silver nanoparticles (PEG–AgNPs). Data are mean  ±  SEM (*n*  =  8 in each group). Statistical analysis by one-way ANOVA followed by Newman–Keuls multiple comparison test.

There was a significant increase in SBP (*p* < 0.0001) in hypertensive (HT) mice treated with saline compared with saline-treated normotensive mice, confirming the occurrence of hypertension (Figure 2A). When compared with the saline-treated group in normotensive mice, intratracheal (i.t.) instillation of PEG–AgNPs resulted in a small but significant increase in SBP (*p* < 0.001). Similarly, PEG–AgNPs administration caused a significant increase in SBP in HT mice when compared with the saline-treated group (*p* < 0.0001). Moreover, SBP was significantly higher in PEG–AgNPs-treated HT mice than in PEG–AgNPs-treated normotensive mice (*p* < 0.0001).

The HR of normotensive mice treated with PEG–AgNPs was slightly higher than in saline-treated mice, although non-statistically significant (Figure 2B). The HR in HT mice exposed to PEG–AgNPs was higher than in HT mice treated with saline (*p* < 0.01) or PEG–AgNPs-treated normotensive mice (*p* < 0.0001; Figure 2B).

### 2.3. Relative Heart Weight

**Figure 3:**
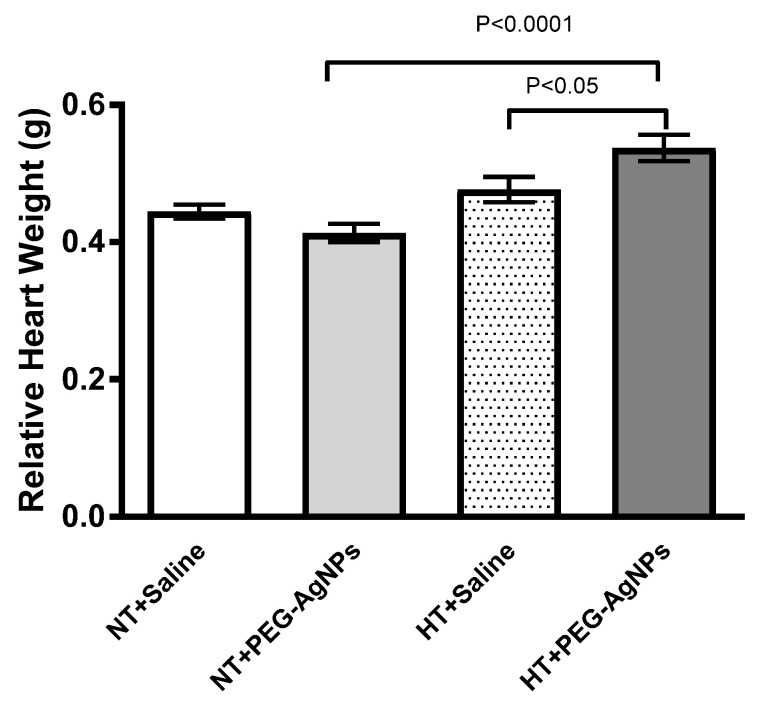
The ratio of heart-weight to body-weight in normotensive (NT) and hypertensive (HT) mice after repeated intratracheal (i.t.) instillation of saline or polyethylene glycol silver nanoparticles (PEG–AgNPs). Data are mean  ±  SEM (*n*  =  8 in each group). Statistical analysis by one-way ANOVA followed by Newman–Keuls multiple comparison test.

The relative heart weight of normotensive mice treated with PEG–AgNPs did not differ statistically from saline-treated mice (Figure 3). PEG–AgNPs significantly increased the relative heart weight in HT mice compared with saline-treated HT mice (*p* < 0.05) or PEG–AgNPs-treated normotensive mice (*p* < 0.0001; Figure 3).

### 2.4. Histopathological Analysis of the Heart

**Figure 4:**
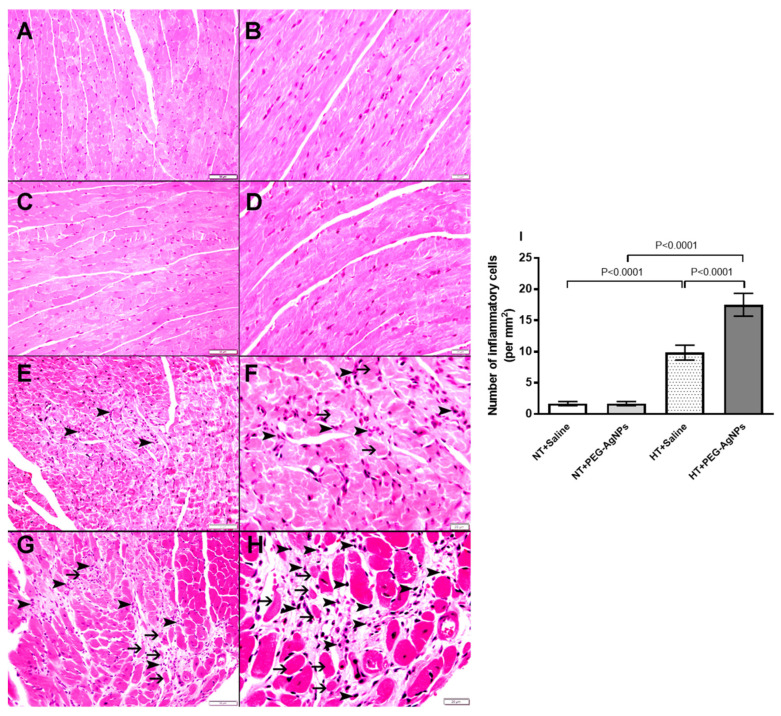
Representative light microscopy sections of heart tissues stained with H&E and obtained from normotensive (NT) and hypertensive (HT) mice after repeated intratracheal (i.t.) instillation of saline or polyethylene glycol silver nanoparticles (PEG–AgNPs). (**A**,**B**) Heart sections obtained from NT mice i.t. instilled with saline showing normal heart architecture and histology. (**C**,**D**) Heart sections obtained from NT mice i.t. instilled with PEG–AgNPs showing normal heart architecture and histology. (**E**,**F**) Heart sections obtained from HT mice i.t. instilled with saline showing the presence of a few spotty areas of cardiomyocyte damage (thin arrow) with inflammatory cells (arrowhead) consisting predominantly of lymphocytes. (**G**,**H**) Heart sections obtained from HT mice i.t. instilled with PEG–AgNPs showing comparatively larger area of cardiomyocyte damage (thin arrow) with increased inflammatory cells (arrowhead) consisting predominantly of lymphocytes than those observed in saline-treated HT mice. (**I**) Quantification of the numbers of inflammatory cells (per mm^2^) in the four studied groups. Data are mean  ±  SEM (*n*  =  6 in each group). Scale bar in (**A**,**C**,**E**,**G**) = 50 µm. Scale bar in (**B**,**D**,**F**,**H)** = 20 µm.

Light microscopy analysis of the heart sections stained with hematoxylin and eosin from normotensive mice treated with saline (Figure 4A,B) or PEG–AgNPs (Figure 4C,D) revealed no cardiac morphological alterations. There were a few spotty focal areas of cardiomyocyte damage with inflammatory cells consisting predominately of lymphocytes in hearts obtained from HT mice i.t. instilled with saline (Figure 4E,F). In the hearts of HT mice exposed to PEG–AgNPs (Figure 4G,H), there were comparatively larger cardiomyocyte damages with increased inflammatory cells infiltration consisting predominantly of lymphocytes than in the hearts of saline-treated HT mice. The analysis of the number of inflammatory cells showed a significant elevation in HT+PEG–AgNPs group compared with HT+saline group or normotensive group treated with PEG–AgNPs (Figure 4I).

**Figure 5:**
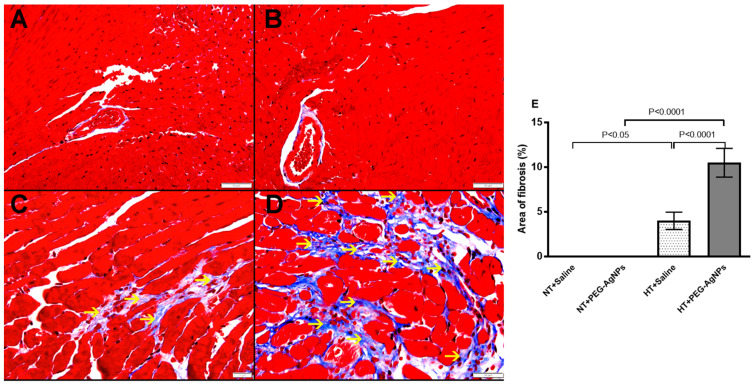
Representative images of Masson trichrome staining of heart tissue obtained from normotensive (NT) and hypertensive (HT) mice after repeated intratracheal (i.t.) instillation of saline or polyethylene glycol silver nanoparticles (PEG–AgNPs). (**A**) The heart sections from NT+saline group showed normal hearts. (**B**) The heart sections from NT+PEG–AgNPs group showed normal hearts. (**C**) The heart sections from HT+saline group showed focal minute area of interstitial fibrosis (thin arrow). (**D**) The heart sections from HT+PEG–AgNPs group showed focal area of interstitial fibrosis (thin arrow). (**E**) Quantification of the area of fibrosis (%) in the four studied groups. Data are mean ± SEM (*n*  =  6 in each group). Scale bar in (**A**–**D**) = 50 µm.

Masson trichrome staining revealed the presence of interstitial fibrosis in HT + saline and HT + PEG–AgNPs-treated mice, and the area of fibrosis was significantly increased in HT+PEG–AgNPs group as compared to HT+saline group or normotensive group treated with PEG–AgNPs (Figure 5).

### 2.5. Lactate Dehydrogenase (LDH) and Creatine Kinase-MB (CK-MB) Activities, and Brain Natriuritic Peptide (BNP) Concentration in Heart

**Figure 6:**
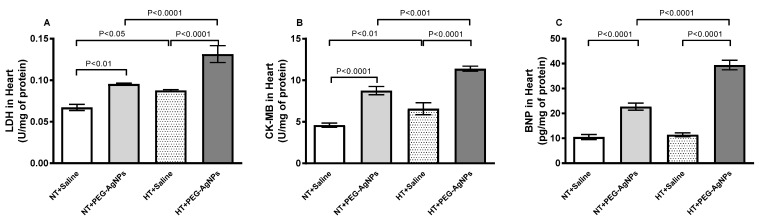
Lactate dehydrogenase (LDH; (**A**)) and creatine kinase-MB (CK-MB; (**B**)) activities and brain natriuritic peptide (BNP; (**C**)) concentration in heart tissues of normotensive (NT) and hypertensive (HT) mice after repeated intratracheal (i.t.) instillation of saline or polyethylene glycol silver nanoparticles (PEG–AgNPs). Data are mean  ±  SEM (*n*  =  6–8 in each group). Statistical analysis by one-way ANOVA followed by Newman–Keuls multiple comparison test.

Figure 6 shows that the activities of LDH and CK-MB and the concentration of BNP were significantly increased in the heart homogenates of normotensive mice given PEG–AgNPs compared with those treated with saline (*p* < 0.0001–*p* < 0.01). Additionally, heart homogenates obtained from HT mice treated with PEG–AgNPs had significantly increased activities of LDH and CK-MB and the concentration of BNP compared with HT mice treated with saline (*p* < 0.0001) or PEG–AgNPs-treated normotensive mice (*p* < 0.0001–*p* < 0.001).

### 2.6. Endothelin-1, P-Selectin, Vascular Cell Adhesion Molecule-1 (VCAM-1), and Intercellular Adhesion Molecule-1 (ICAM-1) Concentrations in Heart

**Figure 7:**
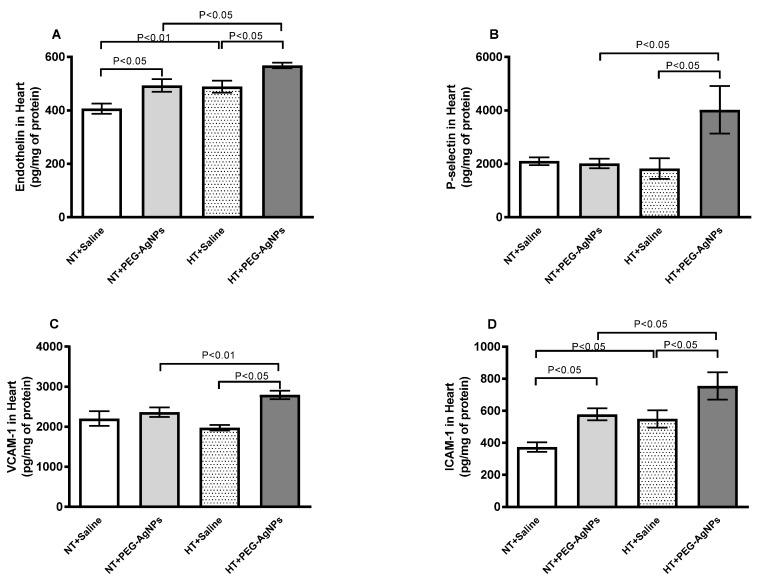
Endothelin (**A**), P-selectin (**B**), vascular cell adhesion molecule-1 (VCAM-1, (**C**)), and intercellular adhesion molecule-1 (ICAM-1, (**D**)) concentrations in heart tissues of normotensive (NT) and hypertensive (HT) mice after repeated intratracheal (i.t.) instillation of saline or polyethylene glycol silver nanoparticles (PEG–AgNPs). Data are mean  ±  SEM (*n*  =  6–8 in each group). Statistical analysis by one-way ANOVA followed by Newman–Keuls multiple comparison test.

Figure 7A–D illustrates that endothelin, P-selectin, VCAM-1, and ICAM-1 concentrations in heart homogenates of HT mice treated with PEG–AgNPs were significantly higher when compared with saline-treated HT group (*p* < 0.05) or PEG–AgNPs-treated normotensive group (*p* < 0.01–*p* < 0.05).

### 2.7. Tumor Necrosis Factor-α (TNFα) and Interleukin-6 (IL-6) Concentrations in Heart

**Figure 8:**
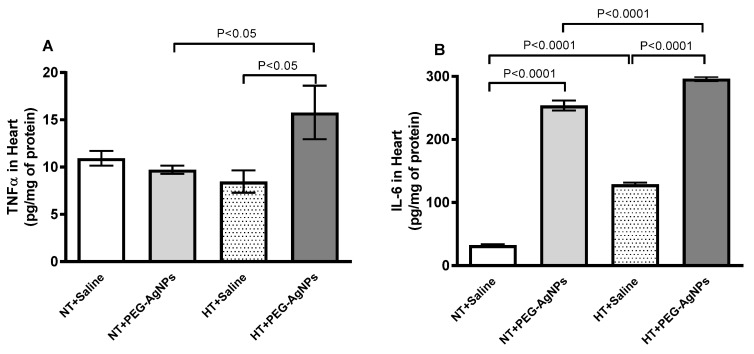
Tumor necrosis factor-α (TNFα; (**A**)) and interleukin-6 (IL-6; (**B**)) concentrations in heart tissues of normotensive (NT) and hypertensive (HT) mice after repeated intratracheal (i.t.) instillation of saline or polyethylene glycol silver nanoparticles (PEG–AgNPs). Data are mean  ±  SEM (*n*  =  7–8 in each group). Statistical analysis by one-way ANOVA followed by Newman–Keuls multiple comparison test.

The effects of PEG–AgNPs on markers of inflammation in the hearts of normotensive and HT mice are depicted in Figure 8. The concentrations of TNF-α and IL-6 in heart homogenates of HT mice treated with PEG–AgNPs were significantly higher than in saline-treated HT mice (*p* < 0.0001–*p* < 0.05) or PEG–AgNPs-treated normotensive mice (*p* <0.0001– *p* < 0.05). Moreover, the concentrations of IL-6 were increased significantly in normotensive mice given PEG–AgNPs versus those treated with saline (*p* < 0.0001).

### 2.8. Lipid Peroxidation (LPO), Reduced Glutathione (GSH), Oxidized Glutathione (GSSG), and Total Nitric Oxide (NO) Concentrations in Heart

**Figure 9:**
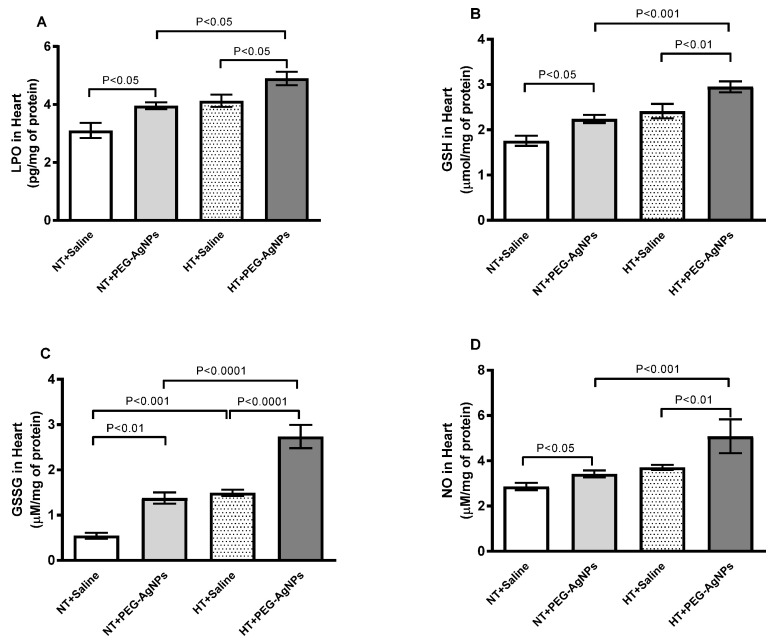
Lipid peroxidation (LPO; (**A**)), reduced glutathione (GSH; (**B**)), oxidized glutathione (GSSG; (**C**)), and total nitric oxide (NO; (**D**)) concentrations in heart tissues of normotensive (NT) and hypertensive (HT) mice after repeated intratracheal (i.t.) instillation of saline or polyethylene glycol silver nanoparticles (PEG–AgNPs). Data are mean  ±  SEM (*n*  =  6–8 in each group). Statistical analysis by one-way ANOVA followed by Newman–Keuls multiple comparison test.

The effects of PEG–AgNPs on markers of oxidative and nitrosative stress in the hearts of normotensive and HT mice are represented in Figure 9. LPO, GSH, GSSG, and total NO concentrations were significantly higher in normotensive mice given PEG–AgNPs versus those treated with saline (*p* < 0.01–*p* < 0.05). Furthermore, HT mice exposed to PEG–AgNPs had significantly higher indices than HT mice treated with saline (*p* < 0.0001–*p* < 0.05) or PEG–AgNPs-treated normotensive mice (*p* < 0.0001–*p* < 0.05).

### 2.9. DNA Damage in Heart

**Figure 10:**
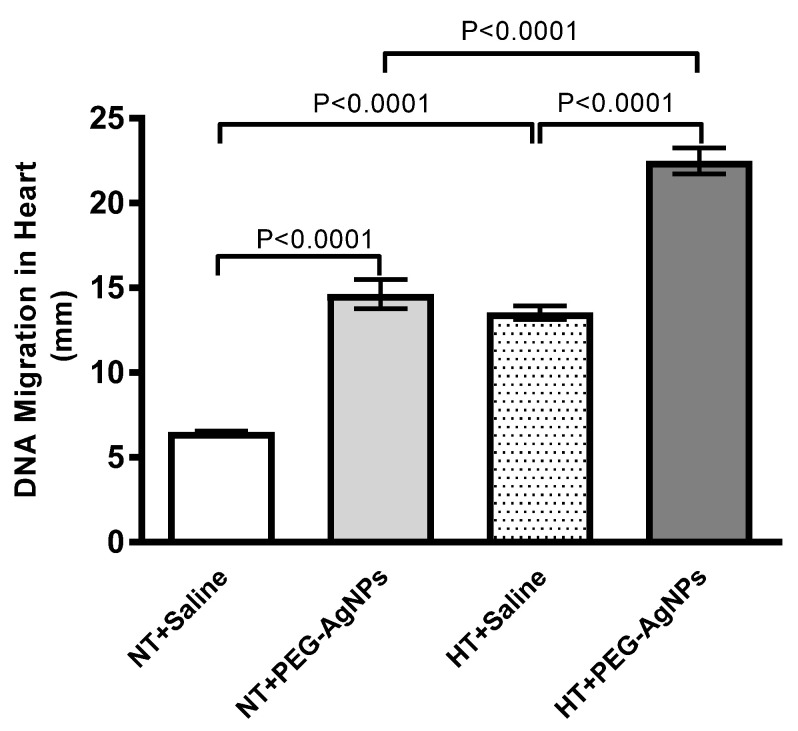
DNA damage, assessed by COMET assay, in heart tissues of normotensive (NT) and hypertensive (HT) mice after repeated intratracheal (i.t.) instillation of saline or polyethylene glycol silver nanoparticles (PEG–AgNPs). Data are mean  ±  SEM (*n*  =  5 in each group). Statistical analysis by one-way ANOVA followed by Newman–Keuls multiple comparison test.

As shown in Figure 10, the i.t. instillation of PEG–AgNPs resulted in a significant increase in DNA injury compared with saline-treated normotensive mice (*p* < 0.0001). Similarly, DNA damage in HT mice exposed to PEG–AgNPs was significantly higher than in HT mice treated with saline (*p* < 0.0001) or PEG–AgNPs-treated normotensive mice (*p* < 0.0001).

## 3. Discussion

As far as we know, this is the first experimental evidence that pulmonary exposure to PEG–AgNPs exacerbates cardiac injury in mice with experimentally induced hypertension. The study found that besides SBP and HR, the cardiac levels of LDH, CK-MB, BNP, adhesion molecules, proinflammatory cytokines, oxidative and nitrosative stress, and DNA damage markers were significantly increased in HT mice exposed to PEG–AgNPs. Similarly, in the latter group of mice, heart histology revealed comparatively larger cardiomyocyte damage with larger area of fibrosis and increased number inflammatory cells.

AgNPs are coated with various materials, including citrate, PVP, or PEG. Here, we examined the effects of 40-nm PEG–AgNPs. Due to its biocompatibility and ability to mask coated particles from opsonins, the latter coating is widely used in the preparation of nanoparticles for pharmaceutical purposes [4,10]. The majority of toxicity studies on AgNPs have been performed on bacteria, cell lines, non-mammalian animal species, or rodent-isolated organs, with still comparatively limited information available from in vivo studies [2,11,12]. In vivo toxicological studies are thus considered critical for correlating the physicochemical properties of nanoparticles with their effects in living systems [2,11,12]. Moreover, they are essential for acquiring more relevant information about human exposure scenarios to nanoparticles. Therefore, in the present study, mice were i.t. instilled with AgNPs. This route of exposure is accurate and allows for exact nanoparticle dosing, as mice are obligate nasal breathers who filter the majority of inhaled particles [13,14]. Moreover, several studies have reported that lung exposure to AgNPs can occur in industrial settings, such as AgNP manufacturing plants, where the concentration of AgNPs in the air increases during production [2,15,16]. Furthermore, pulmonary AgNP exposure is a possible route to aerosolized AgNPs used in sprays, nebulizers, deodorants, and disinfectants. AgNPs are being studied in the field of inhalation therapy in relation to airway allergic inflammation and lung infections [17,18]. Additionally, the 40-nm size of the PEG–AgNPs used allows these nanoparticles to pass through the air–blood barrier and reach several organs, including the heart [2]. We investigated the cardiotoxic effects of repeated AgNP exposure. The latter approach is preferable to single-dose administration as it better simulates human exposure scenarios to nanoparticles. The PEG–AgNPs dose used in this study was 0.5 mg/kg (12.5 µg per mouse of 25-g BW), which is comparable with those reported by others using animal models of i.t. AgNPs administration [19,20]. Since AgNPs are widely used in nanomedicine, it is relevant to evaluate their possible adverse effects in animal model of increased susceptibility, such as hypertensive mice. 

The TEM analysis of the nanoparticles used in this study revealed the presence of homogenous nanospheres of approximately 40 nm in size, and the zeta potential revealed that the particles were neutrally charged. According to our findings, the i.t. instillation of PEG–AgNPs in HT mice resulted in a significant increase in SBP and HR when compared with saline-instilled HT mice and PEG–AgNPs-treated normotensive mice. AgNPs administered as a bolus in isolated heart Langendorff ex vivo model of spontaneously hypertensive rats induces vasoconstriction and increased cardiac contractility [9].

In this study, hearts from PEG–AgNPs-treated HT mice had comparatively larger cardiomyocyte damage with fibrosis and inflammatory cell infiltration than hearts from saline-treated HT group. However, there was no change in heart histology in normotensive mice treated with PEG–AgNPs. The latter findings imply that heart morphological changes are only observed in animals with increased susceptibility, namely, HT mice. The lack of change in heart histology in normotensive mice does not exclude the possibility of ongoing biochemical changes in normotensive mice heart. It has been recently reported that pulmonary exposure to cerium oxide nanoparticles did not change the histology of various organs, including the heart, but caused inflammation and oxidative stress in tissue homogenates [21]. Moreover, we found that the relative ratio of heart-weight to body-weight in HT mice exposed to PEG–AgNPs was significantly higher than in HT mice instilled with saline or PEG–AgNPs-treated normotensive mice. The latter is indicative of cardiac hypertrophy, which is aggravated in HT mice following the exposure to PEG–AgNPs. The exposure of hypertensive mice and rats to particulate air pollution has been reported to cause an increase in heart weight and cardiac dysfunction [22,23]. Additionally, our data show significant increments in cardiac enzyme activities of the cell damage marker, LDH and cardiomyocyte injury marker, CK-MB in HT mice given PEG–AgNPs compared HT mice instilled with saline or PEG–AgNPs-treated normotensive mice, which may be attributed to the myocardial membrane damage [24]. Moreover, our data show that the concentration of BNP, a well-established marker of cardiac dysfunction, was elevated in the heart tissue of HT mice treated with PEG–AgNPs compared with saline-treated HT group or PEG–AgNPs-treated normotensive group. It has been demonstrated that inhalation particulate matter in mice induce a significant increase in the concentration of BNP in heart tissue homogenates [25]. 

Our findings revealed a significant increase in endothelin-1 in heart homogenates of HT mice exposed to PEG–AgNPs when compared with HT mice instilled with saline or PEG–AgNPs-treated normotensive mice. Endothelin-1 production was previously reported to be increased in hypertrophied rat hearts and was linked to pressure overload [26,27]. It has also been shown that endothelin-1 overexpression causes the heart to develop dilated cardiomyopathy and increases in the expression of pro-inflammatory cytokines [28]. In this study, we have also measured three adhesion molecules in heart homogenates: P-selectin, VCAM-1, and ICAM-1. These molecules are found on membrane-activated cells, such as endothelial cells, leukocytes, and platelets, and are recognized endothelial injury biomarkers [29]. Moreover, various studies have reported the upregulation of adhesion molecules both in vitro (cardiac cells) and in vivo (rat heart) under pathophysiological conditions, such as myocardial injury or hypertension [30,31]. Our data revealed a significant increase in P-selectin, VCAM-1, and ICAM-1 in heart homogenates of HT mice exposed to PEG–AgNPs compared with HT mice exposed to saline or PEG–AgNPs-exposed normotensive mice. Cardiomyocytes and endothelial cells are located in near proximity and communicate via the secretion of paracrine signals and through direct cell-to-cell contact [32]. In the myocardium, endothelin-1 can act in both autocrine and paracrine ways [33,34]. It is able to bind to endothelin A receptors on cardiomyocytes and endothelin B receptors on cardiac endothelial cells [33,34]. When it binds to endothelin B receptors, it triggers the release of other signaling molecules, such as NO rather than inducing vasoconstriction [33,34]. On the other hand, when binding to the endothelin A receptors on cardiomyocytes, endothelin-1 induces cardiomyocyte constriction, as observed when vascular smooth muscle cells are treated with endothelin-1 [33,34]. There is a large body of evidence demonstrating the important role played by endothelin-1 in the progression of cardiac dysfunction [33,34,35]. It has been reported that patients with cardiac failure display augmented plasma endothelin-1 concentrations and expression of myocardial endothelin receptors, and the degree of the elevations correlates with disease severity [33,34,36]. Moreover, it has been reported that exposure to particulate air pollution induces endothelin-1 release, which modulates extravascular effects on the heart, adversely affecting cardiac function [35]. The role of endothelin-1 as a mediator of inhaled particulate air pollution-induced cardiac dysfunction is supplementary supported by the therapeutic effectiveness of certain endothelin receptor antagonists [35]. 

It is well known that pulmonary nanoparticle exposure causes cardiac inflammation and oxidative stress [2,3]. The latter effects were explained by the occurrence of lung inflammation and oxidative stress caused by inhaled nanoparticles, which can affect the cardiovascular system and/or the passage of lung-deposited nanoparticles through the alveolar–capillary barrier and their direct interaction with cardiac tissue [2,3,37]. Several in vitro studies have found that AgNPs cause cardiomyocytes and endothelial cell injury and dysfunction [38,39]. Moreover, studies have reported that pulmonary exposure to PVP-coated or citrate-coated AgNPs causes vascular dysfunction and exacerbation of cardiac ischemia-reperfusion injury, cardiac inflammation, and oxidative stress [20,40]. However, the in vivo effects of PEG–AgNPs on cardiac inflammation and oxidative stress in normotensive and HT mice have never been studied. Therefore, to gain insight into the mechanisms of action of PEG–AgNPs in the heart of HT and normotensive mice, we measured markers of inflammation and oxidative and nitrosative stress in heart tissue homogenates. Our data showed that the proinflammatory cytokines TNF-α and IL-6, lipid peroxidation marker LPO, and free radical scavengers GSH and GSSG and total NO were significantly higher in PEG–AgNPs-exposed HT mice than saline-treated HT and PEG–AgNPs-exposed normotensive mice. It is well established that both oxidative stress and inflammation are increased in cardiovascular dysfunction [41,42]. Oxidative stress induces myocardial tissue damage and inflammation, contributing to heart dysfunction development [41,42]. Sequentially, inflammation triggered by oxidative stress or the direct effect of the translocated nanoparticles induces tissue injury, which in turn, aggravates oxidative stress [2,41]. Our data demonstrated the occurrence of both inflammation and oxidative stress, however, additional work is required to differentiate between the effects of inflammation and oxidative stress in relation to the observed toxicity of AgNPs on the heart. The latter would involve targeting oxidative stress and inflammation by using antioxidants or anti-inflammatory agents to disrupt this vicious circle. 

The potentiation of cardiac oxidative stress and inflammation in HT mice exposed to PEG–AgNPs corroborate our heart histology results of PEG–AgNPs-treated HT mice, which showed comparatively larger cardiomyocyte damage with fibrosis and inflammatory cells infiltration when compared with the other studied groups. A recent study reported an increase in TNF-α, IL-6, LPO, GSH, and NO in the hearts of mice given a single dose of PVP-coated or citrate-coated AgNPs [20]. The production of a variety of reactive oxygen and nitrogen oxide species, which play an important role in pathophysiological conditions affecting the heart, characterizes oxidative and nitrosative stress [43]. The latter causes DNA, lipid, and protein injury, resulting in cell dysfunction and death [43]. Our data showed that the i.t. instillation of AgNPs caused a significant increase in DNA injury when compared with control normotensive mice. This effect can be attributed to the nanoparticles’ oxidative and nitrosative stress. Interestingly, when HT mice were exposed to PEG–AgNPs, DNA damage was significantly exacerbated compared with HT mice treated with saline or PEG–AgNPs-treated normotensive mice. The lack of histological changes in the hearts of PEG–AgNPs-treated normotensive mice is consistent with the findings of Ferdous et al. [20] who demonstrated that PVP–AgNPs and citrate–AgNPs cause cardiac oxidative stress and DNA damage but have no effect on heart histology. This could imply that the morphological changes in the heart are only visible after repeated long-term exposure to these nanoparticles and/or in animal models with pre-existing cardiovascular diseases, such as hypertension.

This study aims to assess whether and to what extent pulmonary exposure to PEG–AgNPs exacerbates cardiac injury in mice with experimentally induced hypertension rather than to determine whether the pulmonary deposited silver was in ionic form. Nonetheless, additional experiments using the same protocol and assessing the effects of silver acetate as the source of silver ions (Ag^+^) at 1.55-mg/kg BW (equivalent to the dose of 1 mg/kg of PEG–AgNPs) revealed some differences in the endpoints measured between the effects of Ag^+^ and those of PEG–AgNPs in normotensive and HT mice (Supporting Information Table S1). These findings are consistent with recent studies that show differences in the impact of AgNPs and Ag^+^ when intravenously or i.t. administered into mice [20,44].

The present study has some limitations, including not evaluating additional routes of exposure, such as intravenous administration, not investigating the gender effects of these nanoparticles, and not using more techniques to detect inflammatory and oxidative stress markers, such as Western blot or immunohistochemistry. Another limitation is the lack of quantification of silver in the heart to understand whether the effects were actually induced by silver, regardless of the ionic or particulate form.

## 4. Materials and Methods

### 4.1. PEG–AgNPs

Suspensions of 40.6 ± 3.8-nm PEG-coated AgNPs (BioPure^TM^, Havant, UK) were obtained from nanoComposix (San Diego, CA, USA). We recently assessed the same nanoparticles on experimental thrombosis in HT mice [8]. The silver purity of the nanoparticle stock solution (1 mg/mL) was 99.99%, and the endotoxin content was less than 2.5 EU/mL. The nanoparticle’s surface area was 13.8 m^2^/g. PEG–AgNPs were suspended in sterile 0.9% NaCl. PEG–AgNP suspensions were constantly sonicated for 10 min and vortexed before dilution and intratracheal (i.t.) instillation to reduce nanoparticle aggregation.

### 4.2. PEG–AgNP Characterization by Transmission Electron Microscopy and Surface Charge Analysis by Zeta Potential

PEG–AgNPs were examined using transmission electron microscopy (TEM), as previously reported [8,45]. In brief, the AgNP suspensions were sonicated for 15 min at room temperature (RT). The PEG–AgNP suspensions were placed on a 200 mesh Formvar/Carbon-coated copper grid and allowed to dry at room temperature for an hour. Thereafter, the grids were analyzed, and several images were captured at various magnifications using a Tecnai^TM^, (Casoria, Italy) G^2^ Spirit transmission microscope (FEI Company, Hillsboro, OR, USA).

Regarding the zeta potential analysis of PEG–AgNPs, all samples were diluted to 10% by volume in absolute ethanol, vortexed for 10 min, and then measured for size and zeta potential. The Malvern Zetasizer instrument (Malvern Panalytical, Malvern, UK) and Zetasizer 7.11 software were used for measurement and data processing. All measurements were performed in triplicate and at room temperature.

### 4.3. Treatments

This project was evaluated and approved by the United Arab Emirates University Animal Ethics Committee, and experiments were carried out in compliance with protocols permitted by the committee (approval code#ERA_2019_5876).

BALB/c mice of both sexes, aged 8 to 10 weeks and weighing 20 to 25 g (Animal House of the College of Medicine and Health Sciences, United Arab Emirates University), were housed in light-controlled (12-h light: 12-h dark cycle) and temperature-controlled (23 ± 2 °C) rooms. They had unrestricted access to commercial laboratory chow and unlimited access to tap water. The number of mice used in each group varied depending on the type of experiment performed, ranging from *n* = 5 to *n* = 8. The number of male and female mice utilized for the assessment of the various parameters was similarly distributed among the studied groups. The number of animals utilized in each assessed endpoint is indicated in the figure legends.

A well-validated murine hypertension model was used [22,46,47]. We recently used it to assess the prothrombotic effects of PEG–AgNPs [8]. Mice were given angiotensin II (ANG II, 0.75 mg/kg/day in 0.15 mol/L NaCl and 0.01-N acetic acid) for the duration of the experiments using an osmotic pump (ALZET Osmotic Pump Model 2006, DURECT Corporation, Cupertino, CA). The vehicle was administered to the normotensive controls. ANG II treatment achieves plasma concentrations comparable with those observed in patients with renovascular hypertension [22,48]. The exposure of mice to PEG–AgNPs (0.5 mg/kg) or saline (control) was achieved by i.t. instillation. Mice were anesthetized with isoflurane and positioned supine with their necks extended on an angled board. A Becton Dickinson 24 Gauge cannula was inserted into the trachea through the mouth. The nanoparticles or saline (100 µL) were injected with a sterile syringe, followed by an equal volume of air bolus. PEG–AgNPs (0.5 mg/kg) or saline (control) were i.t. instilled four times (on days 7, 14, 21, and 28 post-angiotensin II or vehicle [control] infusion). On day 29, mice weights and various cardiovascular parameters were measured.

### 4.4. SBP and HR Measurement

SBP and HR were measured using a non-invasive computerized tail-cuff manometry system (ADInstrument, Colorado Springs, CO, USA) [8,49,50]. Animals were trained for 3 days prior to the experiment to avoid technique-induced anxiety. The SBP and HR were measured at the beginning of the experiment and then once a week for the duration.

### 4.5. Heart Histology and Weight

After the measurement of SBP, hearts were collected from euthanized mice. They were cleaned with ice-cold saline, blotted with filter paper, and weighed to determine the ratio of heart-weight to body-weight. Thereafter, for the histology, hearts (*n* = 6 per group) were dissected, casseted, and directly fixed in 10% neutral formalin for 24 h before being dehydrated in increasing concentrations of ethanol, cleared with xylene, and embedded in paraffin. Three-micrometer sections of paraffin blocks were prepared and stained with hematoxylin and eosin [51]. The number of inflammatory cells were analyzed and expressed as the number of inflammatory cells per mm^2^. Moreover, heart sections were stained for presence of fibrosis using Masson trichrome stain following standard techniques [52]. Masson trichrome stains fibrous tissue blue. The percentage of blue-stained fibrotic area in each heart section, stained with Masson trichrome stain, was analyzed and the fibrosis index was calculated and expressed in percentage according to the protocol previously reported [53,54,55].

### 4.6. Quantification of the Concentrations of LDH, CK-MB, BNP, Endothelin-1, P-selectin, VCAM-1, and ICAM-1 in Heart Tissue

The hearts were quickly cleaned with ice-cold PBS (pH 7.4) before being homogenized in 0.1 M phosphate buffer (pH 7.4) containing 0.15 M KCl, 0.1 mM EDTA, 1 mM DTT, and 0.1 mM phenylmethylsulfonyl fluoride at 4 °C. The heart homogenates were spun down (14,000 rpm at 4 °C for 20 min) to remove cellular debris, and the supernatants were frozen at −80 °C pending biochemical analysis [49]. The protein concentration in the homogenates was quantified using the bicinchoninic acid assay. LDH and CK-MB activities were measured spectrophotometrically using kits (Roche, Basel, Switzerland). BNP concentration was measured using ELISA kit from MyBioSource (San Diego, CA, USA). Endothelin-1, P-selectin, VCAM-1, and ICAM-1 were measured using ELISA Kits (R & D Systems, Minneapolis, MN, USA).

### 4.7. Measurement of TNF-α, IL-6, LPO, GSH, GSSG, and Total Nitric Oxide in Heart

As previously reported, heart homogenates were prepared for the determination of oxidative stress markers [49]. TNF-α and IL-6 (R & D Systems, Minneapolis, MN, USA) and GSH and GSSG (Cayman Chemical, Ann Arbor, Michigan) concentrations were determined using the vendors’ protocols. Nitric oxide (NO) was measured using a total NO assay kit that detects the more stable NO metabolites NO_2_^−^ and NO_3_^−^ [8,49]. NADPH-dependent membrane lipid peroxidation was quantified as a thiobarbituric acid reactive substance using malondialdehyde as a standard (Sigma–Aldrich Fine Chemicals, St. Louis, MO, USA).

### 4.8. Evaluation of DNA Injury in the Heart by COMET Assay

In a separate set of mice (*n* = 5), the hearts of normotensive or HT mice exposed to saline or PEG–AgNPs were collected at the end of the exposure protocol and used to measure DNA injury using the COMET assay. This was achieved as previously described [49].

### 4.9. Statistics

The statistical analyses were performed using GraphPad Prism Software (GraphPad Software Inc., La Jolla, CA). Data are presented as mean ± standard error of the mean, and statistical significance was determined using one-way analysis of variance (ANOVA), followed by the Newman–Keuls multiple comparisons test; *p* < 0.05 was considered significant.

## 5. Conclusions

This study provided novel evidence that pulmonary exposure to PEG–AgNPs exacerbates cardiac morphological changes, SBP, HR, cardiac levels of LDH, CK-MB, BNP, adhesion molecules, proinflammatory cytokine, oxidative and nitrosative stress, and DNA damage in a mouse model of hypertension. Our findings highlighted the importance of an in-depth assessment of these nanoparticles before they can be used, particularly in people with pre-existing cardiovascular conditions. 

## Data Availability

The data that support the findings of this study are available from the corresponding author, Abderrahim Nemmar, upon reasonable request.

## Supplementary Materials

The supporting information can be downloaded at: https://www.mdpi.com/article/10.3390/ijms24108890/s1.
