# Supplementary material for: Impact of Intratracheal Administration of Polyethylene Glycol-Coated Silver Nanoparticles on the Heart of Normotensive and Hypertensive Mice
Source: Int J Mol Sci. 2023 May 17;24(10):8890. doi: 10.3390/ijms24108890 (PMC10218879; doi:10.3390/ijms24108890)
Supplement: Supplementary file 1 [file ijms-24-08890-s001.zip › ijms-2300426-supplementary.pdf]

Supplementary Table S1. Systolic blood pressure (SBP), heart rate (HR), relative heart weight and heart tissue homogenate levels of lactate dehydrogenase (LDH), brain natriuretic peptide (BNP), creatine kinase-MB (CK-MB), endothelin-1, P-selctin, vascular cell adhesion molecule-1 (VCAM-1), intercellular adhesion molecule-1 (ICAM-1), tumor necrosis factor-α (TNFα), interleukin-6 (IL-6), lipid peroxidation (LPO), reduced glutathione (GSH), oxidized glutathione (GSSG) and total nitric oxide (NO) and DNA damage in normotensive (NT) and hypertensive (HT) mice after repeated intratracheal (i.t.) instillation of saline or silver acetate

	NT+Saline	NT+Ag$^+$	HT+Saline	HT+Ag$^+$
SBP (mmHg)	86.13 ± 0.95	84.75 ± 3.01	138 ± 1.00$^{\#\#\#\#}$	153.4 ± 3.29$^{****\Delta\Delta\Delta\Delta}$
HR (bpm)	327 ± 6	358 ± 4$^{\#}$	430 ± 10$^{\#\#\#\#}$	457 ± 15$^{\Delta\Delta\Delta\Delta}$
Relative heart weight (g)	0.44 ± 0.01	0.43 ± 0.009	0.47 ± 0.01	0.46 ± 0.03
LDH (U/mg of protein)	0.067 ± 0.003	0.064 ± 0.003	0.087 ± 0.001$^{\#\#\#\#}$	0.109 ± 0.009 $^{**\Delta\Delta\Delta\Delta}$
BNP (pg/mg of protein)	10.52 ± 0.99	10.34 ± 0.56	11.45 ± 0.75$^{\#\#\#\#}$	36.64 ± 1.89 $^{****\Delta\Delta\Delta\Delta}$
CK-MB (U/mg of protein)	4.60 ± 0.24	7.78 ± 0.54$^{\#\#}$	6.58 ± 0.72$^{\#}$	10.64 ± 0.81 $^{***\Delta\Delta}$
Endothelin-1 (pg/mg of protein)	801 ± 64.61	2898 ± 476.9$^{\#}$	1515 ± 54.65	3835 ± 768.3*
P-selectin (pg/mg of protein)	2100 ± 148	1423 ± 153.7	1825 ± 384.9	2128 ± 234.9
VCAM-1 (pg/mg of protein)	2207 ± 186.3	1603 ± 75.83	1978 ± 71.43	2480 ± 260.3 $^{\Delta\Delta}$
ICAM-1 (pg/mg of protein)	3440 ± 20.17	6397 ± 745.6$^{\#\#\#}$	4219 ± 17.54$^{\#\#\#\#}$	8479 ± 360.7$^{****\Delta\Delta\Delta}$
TNFα (pg/mg of protein)	10.93 ± 0.76	24.01 ± 1.61$^{\#\#\#\#}$	8.47 ± 1.18	26.20 ± 0.86****
IL-6 (pg/mg of protein)	32.31 ± 1.53	206 ± 62.9	128.8 ± 2.73	419.1 ± 122.9 $^{**\Delta}$
LPO (pg/mg of protein)	3.1 ± 0.26	6.21 ± 0.42$^{\#\#\#\#}$	4.12 ± 0.21$^{\#}$	6.72 ± 0.19****
GSH (μmol/mg of protein)	1.75 ± 0.11	3.71 ± 0.11$^{\#\#\#\#}$	2.41 ± 0.15$^{\#\#}$	4.56 ± 0.13$^{****\Delta\Delta\Delta\Delta}$
GSSG (μM/mg of protein)	0.54 ± 0.06	2.18 ± 0.28$^{\#}$	1.49 ± 0.06$^{\#\#}$	5.22 ± 0.95$^{****\Delta\Delta\Delta\Delta}$
Total NO (μM/mg of protein)	2.86 ± 0.16	4.42 ± 0.21$^{\#\#\#\#}$	3.71 ± 0.10$^{\#\#\#}$	4.63 ± 0.09***
DNA migration (mm)	6.5 ± 0.04	14.04 ± 0.81$^{\#\#\#\#}$	13.55 ± 0.40$^{\#\#\#\#}$	21.21 ± 0.90$^{****\Delta\Delta\Delta\Delta}$

as the source of Ag+ ions at 1.55 mg/kg BW, which is equivalent to the dose of 1 mg/kg of polyethylene glycol silver nanoparticles (PEG-AgNPs) used in this study [1,2].

Data are mean ± SEM (n = 5-8 in each group). ****P<0.0001, ***P<0.001, **P<0.01 and *P<0.05 compared with HT+Saline group. $^{\Delta\Delta\Delta\Delta}$P<0.0001, $^{\Delta\Delta\Delta}$ P<0.001, $^{\Delta\Delta}$P<0.01 and $^{\Delta}$P<0.05 compared with NT+Ag^{+} group. $^{\#\#\#\#}$ P<0.0001, $^{\#\#\#}$P<0.001, $^{\#\#}$P<0.01, $^{\#}$P<0.05 compared with NT+Saline group. Statistical analysis by one-way ANOVA followed by Newman-Keuls multiple comparison test.

References

1. Recordati, C.; De, M.M.; Bianchessi, S.; Argentiere, S.; Cella, C.; Mattiello, S.; Cubadda, F.; Aureli, F.; D'Amato, M.; Raggi, A.; et al. Tissue distribution and acute toxicity of silver after single intravenous administration in mice: Nano-specific and size-dependent effects. *Part. Fibre Toxicol.* **2016**, *13*, 12.
2. Ferdous, Z.; Al-Salam, S.; Greish, Y.E.; Ali, B.H.; Nemmar, A. Pulmonary exposure to silver nanoparticles impairs cardiovascular homeostasis: Effects of coating, dose and time. *Toxicol. Appl. Pharmacol.* **2019**, *367*, 36–50. https://doi.org/10.1016/j.taap.2019.01.006.
